# Evaluating the outcome of Ambulances Collisions Review Panel in Middle Eastern environment using epidemiological supervised and unsupervised machine learning analyses

**DOI:** 10.5339/qmj.2025.75

**Published:** 2025-08-22

**Authors:** Hassan Farhat, Guillaume Alinier, Rafik Khedhiri, Jerome Ramos, Emna Derbel, Fatma Babay Ep Rekik, Abraham Ranjith, Mohamed Khnissi, Habib Kerkeni, Mohamed Chaker Khenissi, Ali Al-Yafei, Loua Al Shaikh, James Laughton

**Affiliations:** 1 Ambulance Service, Hamad Medical Corporation, PO Box 3050, Doha, Qatar; 2 Faculty of Medicine “Ibn El Jazzar”, University of Sousse, 4000, Sousse, Tunisia; 3 Faculty of Sciences, University of Sfax, 3000, Sfax, Tunisia; 4 School of Health and Social Work, University of Hertfordshire, Hatfield, UK; 5 Weill Cornell Medicine-Qatar, Doha, Qatar; 6 Faculty of Health and Life Sciences, Northumbria University, Newcastle upon Tyne, UK *Email: hassen.farhat@gmail.com

**Keywords:** Ambulance collision, ambulance safety, emergency medical services, corrective measures, machine learning

## Abstract

**Background:**

Ambulance collisions pose a significant occupational risk to personnel, patients, and the public. Despite ongoing efforts to improve safety measures, the complex nature of emergency response operations continues to pose challenges in reducing collision risks.

**Objective:**

This study investigates the role of the dedicated Vehicle Collisions Review Panel at Hamad Medical Corporation Ambulance Service (HMCAS) in identifying, understanding, and managing risks associated with ambulance collisions.

**Methods:**

A retrospective quantitative analysis of HMCAS ambulance collision records from 2023 was conducted using descriptive and bivariate analyses, along with supervised and unsupervised machine learning (ML) techniques – including multinomial logistic regression (MLR), decision tree (DT) analysis, association rule mining (ARM), and time series forecasting – to uncover hidden patterns, predictive insights, and future projections.

**Results:**

A total of 131 ambulance collisions were analyzed. The majority of incidents involved emergency urban ambulances. MLR and DT achieved prediction accuracies of 41% and 35%, respectively. ARM revealed significant association between daytime incidents, normal road conditions, and the absence of patient involvement. Time series forecasting predicted a gradual increase followed by stabilization in collision incidents.

**Conclusion:**

This study highlights the crucial role of a dedicated collision review panel in managing and mitigating ambulance collision risks. ML techniques provided evidence-based support for decision-making. Future research is needed to evaluate the long-term impacts of targeted training programs and safety protocols.

## 1. INTRODUCTION

Ambulance collisions, including road traffic accidents (RTAs), pose a significant challenge to emergency medical services (EMS) worldwide, primarily due to the critical need for rapid response times and the urgent nature of patient care.^1–3^ These incidents not only endanger the safety of EMS personnel and patients but also delay the timely delivery of emergency medical care, potentially increasing patient morbidity and mortality. The United Nations, the European Union, and the International Transport Forum define an RTA as any accident involving at least one road vehicle in motion on a public road or private road to which the public has the right of access, resulting in at least one injury or fatality.^4,5^ According to Qatar’s official authorities, 168 RTA-related deaths were registered in 2023, representing 5.3 deaths per 100,000 person.^6^ Ambulance collisions registered more than that, which included collisions such as those occurred while parking, when already parked, or a collision with a fixed object in confined spaces. In the United States, ambulances are reportedly involved in more than 6,500 collisions annually, with 35% resulting in at least one injury.^7^ Another study identified that the proportion of ambulance-involved RTAs in Saudi Arabia varies from 12% to 44.9%, with the highest proportions in urban areas. Additionally, 90% of ambulance crashes were due to direct collisions, and in 58% of these cases, the collisions were caused by other parties.^8^ Similarly, a study in India identified that factors such as high-speed driving, inadequate driver training, and the use of lights and sirens increase the risk of ambulance-involved RTAs.^9^ Despite the availability of related literature, few studies have explored all types of ambulance collisions beyond RTAs. Moreover, there remains a gap in region-specific analyses, particularly in Qatar, where infrastructural differences can influence the nature and impact of ambulance RTAs. Despite existing safety protocols and regulations in EMS, ambulance collisions continue to occur due to various factors. Traditional safety measures – such as driver training programs, vehicle maintenance protocols, and standard operating procedures – while essential, often fail to fully address the dynamic challenges faced by emergency responders. These challenges include the pressure to respond quickly in time-critical situations, varying road conditions, unpredictable behavior of other road users, and the need to manage patient care simultaneously.

Furthermore, while ambulance collisions, including RTAs, are an inherent risk in emergency operations, it is crucial to proactively address and mitigate these incidents to ensure the highest standards of safe healthcare delivery. Dr. Donald Berwick, a renowned healthcare quality improvement (QI) leader, emphasized the need to “treasure every defect” as an opportunity to understand and improve the system. In this context, each ambulance collision represents a systemic defect, highlighting a critical area in healthcare that requires thorough investigation and improvement to mitigate associated risks. Research from Middle Eastern and worldwide EMS organizations has indicated that many ambulance collisions are attributable to behavioral factors.^9–11^ Therefore, implementing an ambulance collision review process within the EMS systems is essential in managing and reviewing such incidents. This process would review and analyze the temporal and spatial distributions and injury patterns of collision data, identify patterns and contributing factors, and develop evidence-based strategies to reduce the incidence of such events. The review would focus on behavioral and system-related factors contributing to these events, and would then determine the appropriate corrective measures and proactively reduce the reoccurrence of similar incidents. There is also a gap in region-specific analyses, particularly in areas such as Qatar, where infrastructural characteristics can influence the nature and impact of ambulance collisions.

The aim of this study is to demonstrate the importance of a dedicated collision review panel in managing and mitigating risks associated with ambulance operations in EMS – by examining the ambulance collisions reviewed by the panel implemented at Hamad Medical Corporation Ambulance Service (HMCAS) in Qatar – and the role of artificial intelligence computing techniques in decision making for corrective measures.

## 2. METHODS

### 2.1. Study design and setting

This study was a retrospective quantitative analysis of ambulance collision records from HMCAS in 2023. The HMCAS Quality and Patient Safety (QPS) Department coordinates a weekly Vehicles’ RTA and Damage Panel to investigate HMCAS ambulance collisions weekly (Every Monday). With the assistance of the HMCAS QPS Department, the panel collects all relevant information, explores the incident’s circumstances, identifies the lessons learned and any corrective measures, and issues recommendations to mitigate future occurrences. In the case of a collision where the police identify the HMCAS driver as being at fault according to the Qatar road traffic code, or the collision could be avoided despite the crew not being determined at fault by the police, the HMCAS QPS Department invites the involved crew to the panel review. All the information is maintained in electronic system. In HMCAS, emergency and non-emergency medical response units are driven by licensed paramedics who receive three weeks of specific training on emergency vehicle defensive driving, coordinated by the HMCAS Training Department and are then assessed theoretically and practically before being allowed to drive emergency response vehicles.^12,13^ Supervisors and managers with an ambulance paramedic (AP) and critical care paramedic (CCP) scope of practice can also respond to emergency calls when needed.^14^

### 2.2. Participants

This study included all collisions involving road response vehicles operated by HMCAS, including collisions while responding to emergency calls, patient transport to a healthcare facility, transit to standby locations, and during parking maneuvers. The Vehicles’ RTA and Damage Panel members must have reviewed these collision incidents, and the HMCAS crew involved must have been invited to the panel review. Incidents involving bicycles or aeromedical transport operations were excluded from the analysis. Additionally, ambulance collisions where the initial police report identified the HMCAS staff were not at fault and were not interviewed by the Vehicles’ RTA and Damage Panel members were also excluded – unless the collision could have been avoided despite the crew not being determined at fault by the police. In such cases, the staff were included and interviewed to determine the root cause and to identify potential content for inclusion in the ambulance defensive driving course.

### 2.3. Variable and data analysis

***2.3.1. Descriptive, bivariate analysis***

Various variables related to ambulance collisions were considered. These variables included patient involvement, ambulance status, ambulance type, and the location of the incidents. Descriptive, bivariate, and multivariate analyses were conducted. The counts and percentages for each variable, grouped by time of day, were determined to summarize the characteristics of the HMCAS collisions. Fisher’s exact tests were conducted to examine the associations between the categorical variables and time of day, with *p*-values and confidence intervals (CIs). A Shewhart control chart was used to display the weekly proportions of ambulance collisions reviewed by the panel relative to the total weekly collisions recorded.

***2.3.2. Multinomial logistic regression and decision tree analysis***

A combination of supervised machine learning (ML) techniques was employed to analyze and predict the actions of the ambulance collision panel based on various factors within a medical emergency response context. Supervised ML is a technique where an algorithm learns from a dataset that includes input and output labeled data. The algorithm uses these labeled examples to learn the relationship between inputs and outputs to predict the results for new, unseen data accurately.^15^ Data preparation and categorical variables conversion to factors were performed. The multinomial logistic regression (MLR) model was used to predict the actions of ambulance collision panels using predictors such as the time of day of collision, response unit status, ambulance type, location (urban vs. rural), and whether a patient was involved or not. The MLR produced odds ratios (ORs), enabling interpretation of how each predictor influenced the likelihood of different actions. A decision tree (DT) model was employed to create a hierarchical structure of decision rules.^16^ The DT offered an interpretable, visual representation of the decision-making process – where transparency in decision-making is crucial. Both models were evaluated using predictive accuracy. The DT model was validated using a confusion matrix, which simultaneously assessed the accuracy, sensitivity, specificity, and precision of the model.

***2.3.3. Association rule mining***

Association rule mining (ARM) is a rule-based unsupervised ML technique that reveals the relationships and connections between variables.^17^ Unsupervised ML is an algorithm that analyzes data without labels or predefined categories.^15^ The algorithm explores the data to find the hidden patterns, groupings, or structures without being given the correct answers.^15^ ARM was employed to identify the factors frequently associated with ambulance collisions. The Apriori algorithm, an unsupervised ML algorithm, was employed to determine most frequently appearing items and the relationships that are significant.^18^ It operates on the principle of identifying frequent patterns and sets of variables that occur together often in the dataset. The generated rules were sorted based on the strength of the association. The key rules describing the association between the “staff involvement” variable and other variables were visualized using the network and parallel coordinates plots, which visualize and show the analysis results of association rules involving multiple variables. To ensure a robust validation of the ARM in filtering out weak or spurious associations, a minimum support threshold of 0.01 (1%) was set to ensure statistical significance by considering only patterns that appeared in at least 1% of cases. Additionally, a high confidence threshold of 0.8 (80%) was also set to ensure strong relationships between identified factors.

***2.3.4. Time series forecasting analysis***

Time series forecasting statistical model methods were employed to predict future ambulance collisions based on historical data. An autoregressive integrated moving average (ARIMA) model was fitted to the time series data to forecast future values of weekly ambulance collision counts. The ARIMA model uses past values in the time series (autoregressive component) and previous error terms (moving average component) to forecast future values.^19^ It also makes the data more consistent by removing trends and repeating patterns, which helps predict future values.^19^ The ARIMA algorithm automatically validates itself by selecting the best-fitting parameters through the Akaike Information Criterion, as a quality check that balances model accuracy with simplicity.^20^ A Shewhart X-bar control chart was used to monitor the mean of the forecasted values and identify potential deviations from expected patterns. The forecasted weeks were highlighted on the Shewhart control chart to distinguish them from the historical data and illustrate future trends and possible deviations.

### 2.4. Ethical approval

This study was approved by the Hamad Medical Corporation Ambulance Service Group Research and Oversight Committee as an audit with reference number AS 2024-320.

## 3. RESULTS

**Figure 1:**
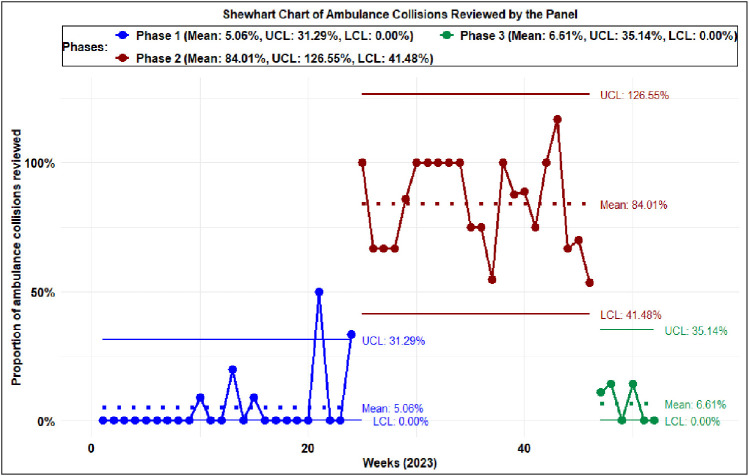
Shewhart control chart of the weekly proportion of ambulance collisions reviewed by the Hamad Medical Corporation Ambulance Service Vehicles’ Damage and Road Traffic Accident Panel in 2023.

In 2023, 131 (38.87%) of 337 ambulance collisions met the inclusion criteria. The remaining 206 ambulance collisions (61.13%) that did not meet the inclusion criteria were unavoidable incidents with minor impacts and were not required to be reviewed by the Vehicles’ RTA and Damage Panel – such as in a rear-end collision while stopping at a red traffic signal. The control charts in Figure 1 show special causes at the end of July, the end of August, the end of September, and the beginning of October. Most of these periods are the peak heat periods in Qatar. The upper control limit (UCL), lower control limit (LCL), and the mean were rephased due to an observed shift, indicating a statistically significant deviation from the baseline data variation pattern.

Tables 1 and 2 and Appendix 1 show the results of descriptive, bivariate, and MLR modeling. The MLR accuracy was equal to 41%. A statistically significant difference was observed in the distribution of incidents across weeks between day and night shifts, suggesting distinct temporal patterns in ambulance collisions. Despite no statistical significance difference (*p* = 0.08), there were more incidents involving patients during daytime operations (29.90%) compared to nighttime operations (15.90%). Emergency urban ambulances were the most frequently involved in collisions, with slightly different proportions in daytime (63.20%) and nighttime (81.80%) incidents. The most common status was “Available”, indicating that collisions mainly occurred when the ambulance was not responding to an emergency call. A statistically significant difference was observed in the exact locations of incidents between day and night shifts (*p* < 0.001), leading to the importance of considering specific environmental factors in safety protocols. Further, the MLR in Table 2 analyzed factors associated with different recommended corrective measures by the review panel following ambulance collisions. For the “Monitoring for One Year” recommendation, ambulances responding to Priority 1 (P1) calls had significantly higher odds ratio (OR = 336, 95% CI: 58.0–1,951) than available ambulances. Priority 1 calls are the ones when an ambulance is dispatched with a light and a siren to a serious emergency call, whereas P2 is one when an ambulance moves without a light and a siren.^21^ Additionally, incidents at traffic signals with green lights were associated with higher odds ratio (OR = 979, 95% CI: 321–2,980) than highway incidents. Similar patterns were observed for the “No Action Required” recommendation, with responding to P1 calls (OR = 108, 95% CI: 19.7–597) and transporting P2 patients (OR = 663, 95% CI: 126–3,495) associated with higher odds. The “Driving Refresher Course” recommendation showed high variability across different factors. This suggests that the factors collected by the panel and considered in the MLR analysis – such as the type of call (emergency vs. non-emergency), the location of the incident, and the ambulance status at the time of collision – are essential in determining the recommended corrective measures. It is noteworthy that the extremely large (> 2,000) or small (< 0.01) ORs indicate issues with model stability or data deficiency in specific categories.

**Figure 2:**
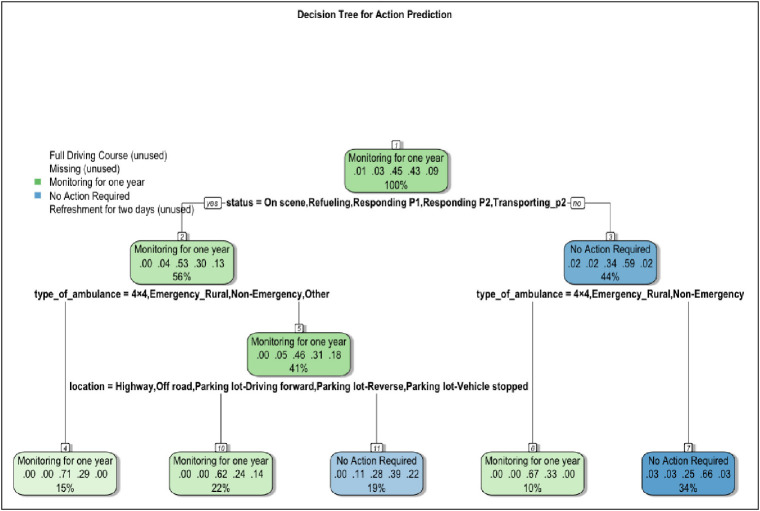
Decision tree plot for predicting the Hamad Medical Corporation Ambulance Service Collision Panel’s corrective measures.

Figure 2 shows the DT plot for predicting the corrective measures decisions of the review panel and the factors influencing these decisions with an accuracy of 35%. At the root node, the primary split is based on the ambulance’s status during the collision. For ambulances on scene, refueling, responding to P1 or P2 calls, or transporting P2 patients, the model predicts “Monitoring for one year” as the most likely action (100% of cases in this branch). For ambulances in other statuses, the tree further splits based on the type of ambulance. Emergency urban ambulances are treated separately from other types (4 × 4, emergency rural, non-emergency, and other). For non-emergency urban ambulances, the model predicts “Monitoring for one year” with a 56% probability, while for emergency urban ambulances, “No Action Required” is predicted with a 44% probability. The DT further refines predictions for non-emergency urban ambulances based on the incident location. For incidents occurring on highways, off-road, or in various parking lot situations (driving forward, reverse, or vehicle stopped), the model predicts “Monitoring for one year” with a 41% probability. This branch splits again into more specific predictions based on exact locations, with probabilities ranging from 15% to 22% for “Monitoring for one year” and 19% for “No Action Required” in certain parking situations. The “Refresher for two days” and “Full Driving Course” actions are marked as unused in this model, indicating they are either rarely recommended or that the current decision tree structure fails to effectively capture the conditions leading to these recommendations. This potentially indicates the effectiveness of the review panel in maintaining control over the behavioral patterns of HMCAS staff, as evidenced by the absence of extreme corrective measures such as “Refresher for two days” and “Full Driving Course”. Overall, the DT indicates that the ambulance’s status at the time of the incident is the most critical factor in determining the recommended action, followed by the type of ambulance and the specific location of the incident, both of which help guide the implementation of appropriate safety measures and corrective actions addressing these factors.

**Figure 3:**
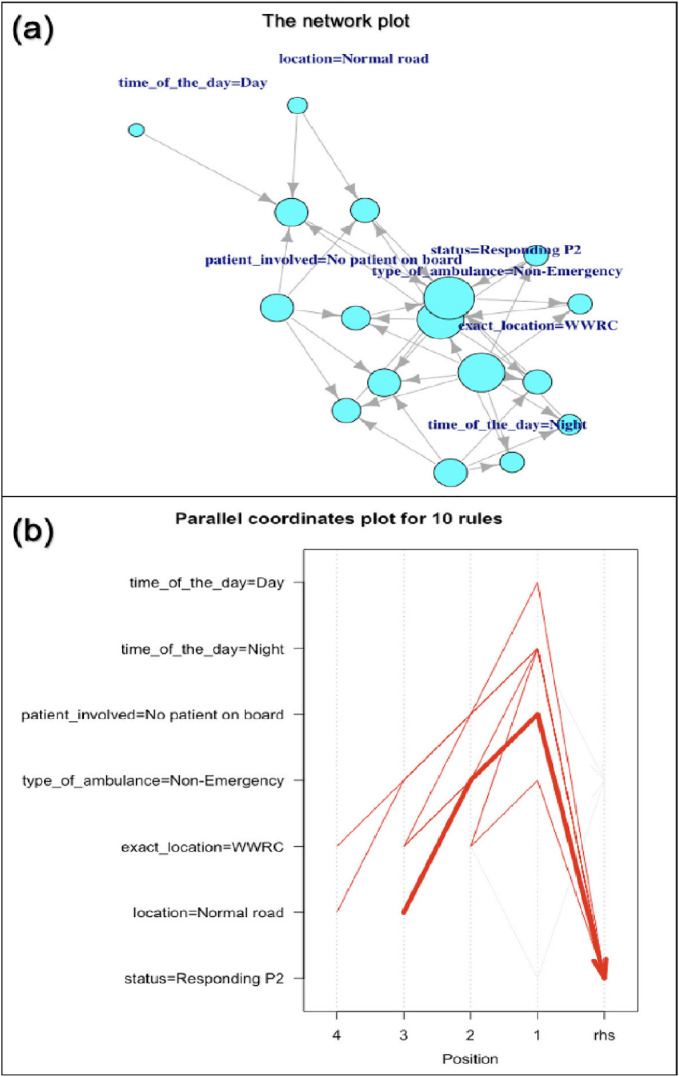
Association rule mining plots of the ambulance collision factors (Hospital2 = WWRC). (a) Network visualization displaying interconnected factors with node size proportional to variable importance and edges representing rule associations. (b) Parallel coordinates plot showing 10 association rules with line thickness indicating rule strength and position indicating variable values.

The network plot in Figure 3a shows the relationships between attributes related to collisions. The nodes represent various attributes, with the size of the nodes indicating their relative importance or frequency in the dataset. The larger nodes – such as “No patient on board”, “Non-Emergency” ambulance type, and “Hospital2” as the exact location – indicate that these occur more frequently. The connections between the nodes indicate the associations between these variables, with arrows showing the direction of these relationships. Notable connections are observed between “time_of_the_day=Day”, “location=Normal road”, and “patient_involved=No patient on board”, indicating a pattern of incidents occurring during the daytime on normal roads without patients on board, which is a good indicator. The parallel coordinates plot in Figure 3b shows ten association rules, with each vertical axis representing a different attribute or condition. The lines connecting the axes represent the rules, and the thickness of the lines indicates their strength or importance. The plot reveals significant associations between various factors, including “time_of_the_day” (both Day and Night), “patient_involved=No patient on board”, “type_of_ambulance=Non-Emergency”, “exact_location=Hospital2”, “location=Normal road”, and “status=Responding P2”. The convergence of lines towards the right side of the plot (rhs) indicates that these factors commonly co-occur in the incidents analyzed.

**Figure 4:**
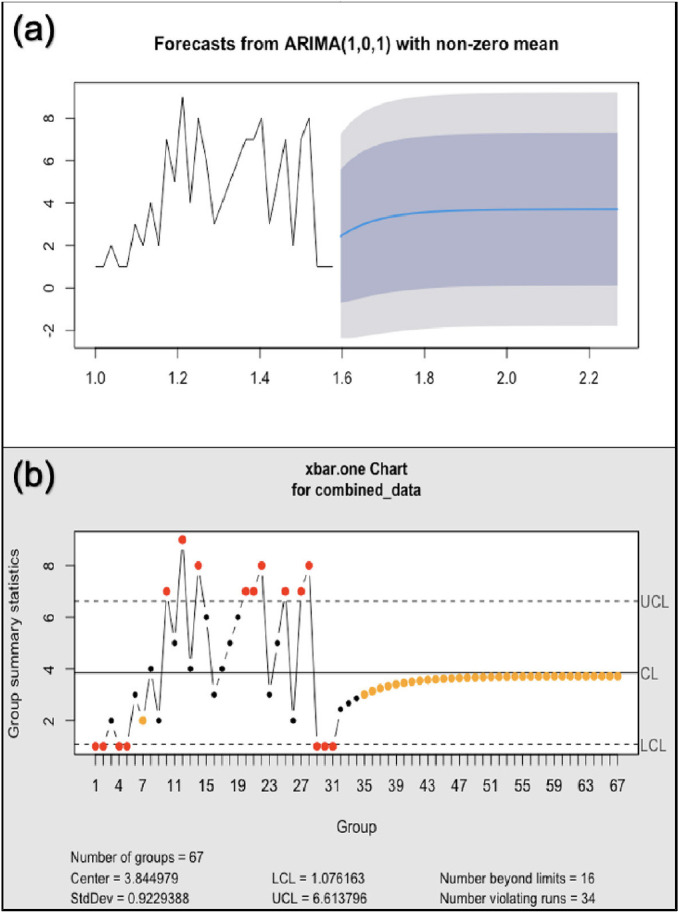
Time series forecasting analysis plots of ambulance collisions in 2023. (a) ARIMA(1,0,1) model forecasts with confidence intervals. (b) X-bar control chart showing process control statistics with upper and lower control limits.

Figure 4a presents the time series forecasting analysis using an ARIMA model. The black line represents the historical data, while the blue line indicates the forecasted values. The shaded areas around the forecast line represent the CIs. The model predicts the series’ gradual increase and stabilization, with widening CIs indicating increasing uncertainty over time. Figure 4b presents a Shewhart control chart (X-bar chart) for process monitoring, providing a better picture of the forecasted data. The chart shows significant variability in the data, with multiple points exceeding the UCL and LCL. The presence of 16 points beyond the control limits and 34 violating runs indicates that the process is not in statistical control. These findings suggest the need for further investigations to identify the sources of these variations in the forecasted data.

## 4. DISCUSSION

This study highlights the important role of a dedicated collision review panel in managing and mitigating risks associated with ambulance collisions in daily EMS operations through the example of the HMCAS Ambulance Collisions Review Panel. Implementing such panels would further enhance patient and staff safety in the high-risk prehospital care environment. This initiative aligns with global efforts to improve EMS safety through systematic review processes.^22^

The analysis of the data collected by the panel revealed distinct temporal patterns in ambulance collisions, with a statistically significant difference in incident distribution between day and night shifts – indicating the need for targeted safety strategies for each operational period. The fewer patient-involved incidents at night (15.90% compared to 29.90% during the day) suggest that night-time operations may be safer for patients, possibly due to reduced traffic and heightened crew alertness – as they are more aware of the higher risk of environmental factors – as demonstrated in other researches.^23,24^ The MLR analysis identified key risk factors associated with different recommended actions following ambulance collisions. Notably, ambulances responding to P1 calls had significantly higher odds of receiving a “Monitoring for One Year” recommendation than available ambulances. The HMCAS Review Panel paid particular attention to accidents occurring during P1 responses – despite their minor aspect – due to their potential impact on the community and staff. Despite the minor damage recorded by these collisions, the HMCAS Collisions Review Panel proactively addressed this issue by incorporating defensive driving training into P1 driving courses. This training is conducted for three weeks, with daily practice on congested roads. No accidents were recorded during these training sessions, suggesting an effective risk mitigation strategy. Additionally, although not directly addressed in the data analysis, community awareness is also crucial in creating a safer environment for emergency vehicle operations.

Further, advanced ML analytical techniques such as DT, ARM, and time forecasting help provide robust reasoning for the panel’s evidence-based decision-making. The DT provided a clear, hierarchical structure for predicting recommended actions based on incident characteristics, enabling straightforward interpretation and implementation of safety protocols. The network plot (Figure 3) shows in-depth relationships between various factors related to ambulance collisions. Connections between daytime incidents, normal roads, and no patient involvement are observed. This was further illustrated by the parallel coordinates plot, which highlighted the frequent co-occurrence of certain factors such as non-emergency ambulances operating in specific locations like Hospital2 and P2 response statuses. Together, these advanced analytical techniques can provide a new dimension to the perspective on risk mitigation and QI in prehospital settings. Consequently, the hierarchical stratification provided by the DT can help EMS organizations worldwide to develop targeted training programs for specific operational scenarios – similar to the training programs recommended by the HMCAS Ambulance Collisions Review Panel in this study – and improve overall safety.

On the other hand, time series forecasting enables anticipation of high-risk periods, allowing for proactive resource allocation and the implementation of additional safety measures during these times. The network plot analysis (Figure 3) can help optimize ambulance route planning, especially for non-emergency transports, to reduce collision risks in high-risk areas or during specific periods. Similar findings have been demonstrated in other studies.^25,26^ By integrating these analytical models into daily operations, EMS providers can perform real-time risk assessments for ambulance collision incidents and make robust data-driven decisions to enhance safety during each mission. The adoption of these advanced analytical techniques and ML models by HMCAS and EMS organizations worldwide would provide another dimension for the continuous improvement culture, enhancing their ability to identify, predict, and mitigate risks associated with ambulance operations.

## 5. LIMITATIONS

This study has several limitations. Firstly, the data analyzed was specific to the HMCAS system in Qatar, which may limit the generalizability of the findings to other EMS systems operating in different geographic regions or under different environmental conditions. Secondly, the retrospective nature of the study relied on the accuracy and completeness of the recorded data in the HMCAS electronic system. Any potential biases or inconsistencies in data collection and reporting could impact the validity of the results. Thirdly, while the study identified associations between various factors and staff-fault ambulance collisions, it did not determine causality. Additional research, including interventional studies, would help determine the causal relationships between the identified factors and ambulance collisions. Another limitation involves potential confounding variables that were not accounted for in the analysis. Factors such as individual driver characteristics, specific road conditions, and other environmental influences may have contributed to ambulance collisions but were not included in the available dataset. Finally, although ML techniques were employed, their performance and accuracy may have been influenced by the quality and scope of the data. Further refinement and validation of the ML models, along with the incorporation of additional data sources, could enhance their predictive capabilities and applicability in real-world scenarios.

## 6. CONCLUSION

This study demonstrated that a dedicated collision review panel, supported by advanced analytical techniques, can enhance safety and QI in prehospital care. Further, the panel can help manage and mitigate risks associated with ambulance operations in EMS. The integration of advanced analytical techniques enhanced the panel’s ability to make evidence-based decisions. These methods identified key risk factors and provided a robust framework for predicting and preventing future incidents. Future research should focus on longitudinal studies to evaluate the long-term impact of these targeted interventions and safety protocols. Additionally, incorporating real-time data analytics and ML models into daily EMS operations may further improve risk assessment and decision-making processes.

## LIST OF ABBREVIATION

**Table:** 

APs	Ambulance Paramedics
ARIMA	Autoregressive Integrated Moving Average
ARM	Association Rule Mining
CCPs	Critical Care Paramedics
CIs	Confidence Intervals
DT	Decision Tree
EMS	Emergency Medical Services
LCL	Lower Control Limit
ML	Machine Learning
ORs	Odds Ratios
QI	Quality Improvement
QPS	Quality and Patient Safety
RTAs	Road Traffic Accidents
UCL	Upper Control Limit

## ACKNOWLEDGEMENT

We thank Mr. Houcine Kharmous and Mr. Hichem Askri for their support on the RTA panel, and Mr. Jeffrey Dillow for his contribution to the post-RTA action follow-up.

## AUTHORS’ CONTRIBUTION

HF: contributed to conceptualization, data analysis, and writing the manuscript. GA, JL, and LS: reviewed the manuscript and supervised the study. RK, FB, ED, JR, and AR: performed the data validation. AG, HK, AAY, and MCK: reviewed the manuscript.

## AVAILABILITY OF DATA AND MATERIAL

The data is available with the first author upon reasonable request and pending approval from the Hamad Medical Corporation Ambulance Service Executive Team.

## CONSENT FOR PUBLICATION

All authors approve the publication of this manuscript.

## COMPETING INTERESTS

The authors have no conflicts of interest to declare.

## PRESENTATIONS

This study was not presented in any event.

## Figures and Tables

**Appendix 1:**
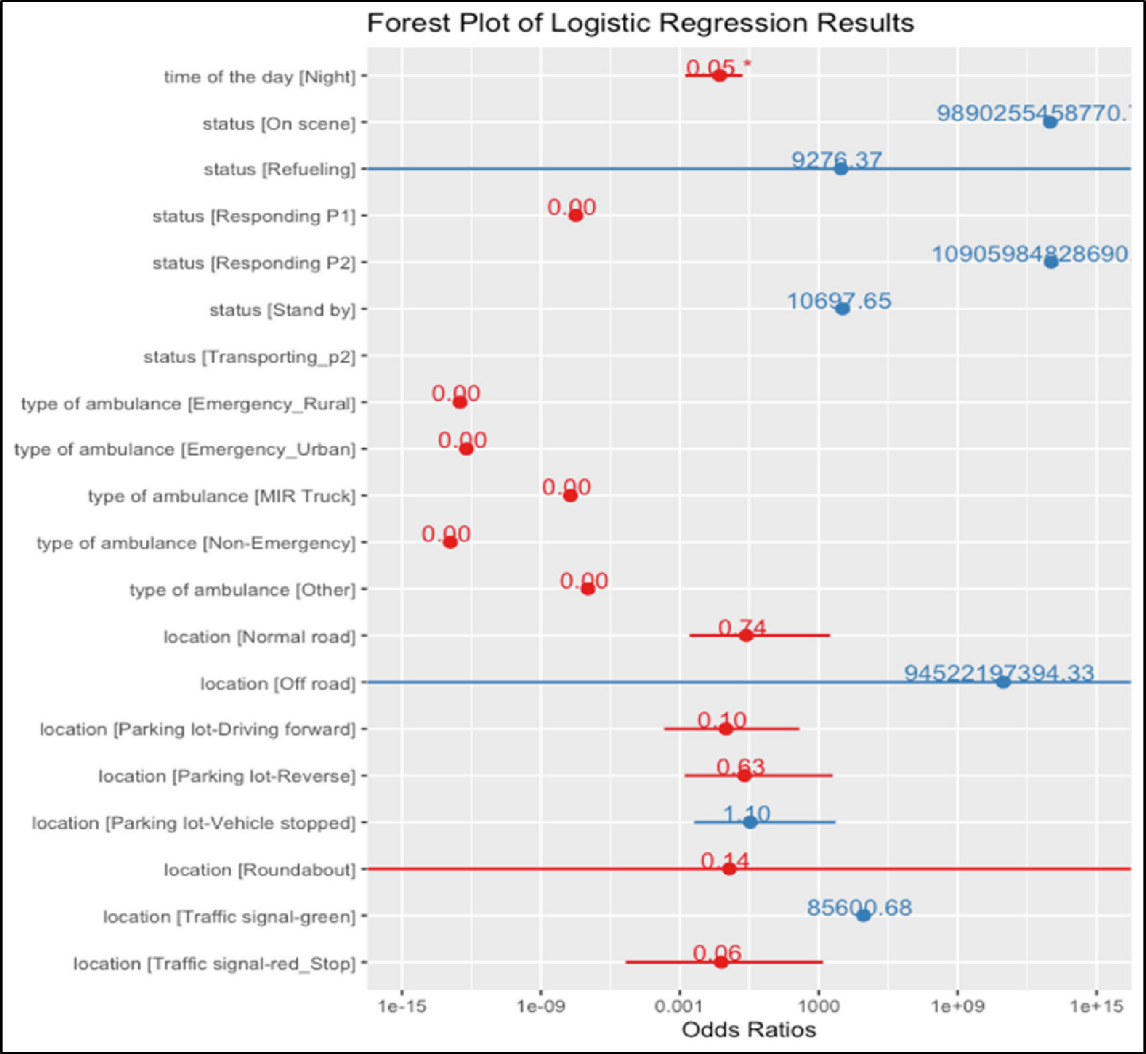
Forest plot of the multinomial logistic regression model odd ratios results.

**Table 1.** Summary statistics of 2023 ambulance collision’s cohort by time of the day.

**Characteristic**	**Day, *n* = 87^1^**	**95% CI^2^**	**Night, *n* = 4 4^1^**	**95% CI^2^**	***p*-value^3^**
**Incident_week**					<0.001
**Staff Nationality**					0.5
Egyptian	1 (1.1%)	0.06%, 7.1%	0 (0.0%)	0.00%, 10%	
Filipino	18 (20.7%)	13%, 31%	6 (13.6%)	5.7%, 28%	
Indian	26 (29.9%)	21%, 41%	10 (22.7%)	12%, 38%	
Jordanian	19 (21.8%)	14%, 32%	15 (34.1%)	21%, 50%	
Tunisian	23 (26.4%)	18%, 37%	13 (29.5%)	17%, 45%	
**Patient**					0.082
No patient on board	61 (70.1%)	59%, 79%	37 (84.1%)	69%, 93%	
Patient on board	26 (29.9%)	21%, 41%	7 (15.9%)	7.2%, 31%	
**Type of the ambulance**					0.2
4×4	9 (10.3%)	5.1%, 19%	1 (2.3%)	0.12%, 14%	
Emergency_Rural	3 (3.4%)	0.89%, 10%	2 (4.5%)	0.79%, 17%	
Emergency_Urban	55 (63.2%)	52%, 73%	36 (81.8%)	67%, 91%	
Major Incident Response Truck	1 (1.1%)	0.06%, 7.1%	0 (0.0%)	0.00%, 10%	
Non-Emergency	17 (19.5%)	12%, 30%	5 (11.4%)	4.3%, 25%	
Other	2 (2.3%)	0.40%, 8.8%	0 (0.0%)	0.00%, 10%	
**Status**					0.3
Available for a call	39 (44.8%)	34%, 56%	14 (31.8%)	19%, 48%	
On scene	11 (12.6%)	6.8%, 22%	10 (22.7%)	12%, 38%	
Refueling	2 (2.3%)	0.40%, 8.8%	0 (0.0%)	0.00%, 10%	
Responding P1	5 (5.7%)	2.1%, 14%	6 (13.6%)	5.7%, 28%	
Responding P2	10 (11.5%)	5.9%, 21%	6 (13.6%)	5.7%, 28%	
Standby	1 (1.1%)	0.06%, 7.1%	1 (2.3%)	0.12%, 14%	
Transporting_p2	19 (21.8%)	14%, 32%	7 (15.9%)	7.2%, 31%	
**Location**					0.3
Highway	0 (0.0%)	0.00%, 5.3%	2 (4.5%)	0.79%, 17%	
Normal road	36 (41.4%)	31%, 52%	18 (40.9%)	27%, 57%	
Off-road	1 (1.1%)	0.06%, 7.1%	0 (0.0%)	0.00%, 10%	
Parking lot-Driving forward	20 (23.0%)	15%, 33%	9 (20.5%)	10%, 36%	
Parking lot-Reverse	8 (9.2%)	4.3%, 18%	5 (11.4%)	4.3%, 25%	
Parking lot-Vehicle stopped	12 (13.8%)	7.6%, 23%	10 (22.7%)	12%, 38%	
Roundabout	2 (2.3%)	0.40%, 8.8%	0 (0.0%)	0.00%, 10%	
Traffic signal-green	3 (3.4%)	0.89%, 10%	0 (0.0%)	0.00%, 10%	
Traffic signal-red_Stop	5 (5.7%)	2.1%, 14%	0 (0.0%)	0.00%, 10%	
**Exact_location**					<0.001
**Corrective measures**					>0.9
Full Driving Course	1 (1.1%)	0.06%, 7.1%	0 (0.0%)	0.00%, 10%	
Missing	2 (2.3%)	0.40%, 8.8%	1 (2.3%)	0.12%, 14%	
Monitoring for one year	40 (46.0%)	35%, 57%	19 (43.2%)	29%, 59%	
No Action Required	37 (42.5%)	32%, 54%	20 (45.5%)	31%, 61%	
Refresher 2 Days	1 (1.1%)	0.06%, 7.1%	0 (0.0%)	0.00%, 10%	
Refresher for two days	6 (6.9%)	2.8%, 15%	4 (9.1%)	3.0%, 23%	

^1^*n* (%); ^2^CI: Confidence interval; ^3^Fisher’s exact test; Pearson’s Chi-squared test.

**Table 2.** Multinomial logistic regression results of 2023 Ambulance Collision Panel’s corrective measures.

**Corrective measures**	**Odds ratio (95% CI)**		***p*-value**
	**OR^1^**	**95% CI^1^**	
**I. Monitoring for one year**			
**Time_of_the_day**			0.8
Day	—	—	
Night	>2,000	—	
**Status**			0.3
Available	—	—	
On scene	>2,000	—	
Refueling	>2,000	—	
Responding P1	336	58.0, 1,951	
Responding P2	>2,000	—	
Standby	0.00		
Transporting_p2	>2,000	1,345, 38,189	
**Type of the ambulance**			0.7
4×4	—	—	
Emergency_Rural	>2,000	—	
Emergency_Urban	0.00	0.00, 0.00	
Major Incident Response Truck	0.00	0.00, 0.00	
Non-Emergency	0.77	0.22, 2.64	
Other	>2,000	—	
**Location**			>0.9
Highway	—	—	
Normal road	0.00	0.00, 0.00	
Off-road	>2,000	—	
Parking lot-Driving forward	0.00	0.00, 0.00	
Parking lot-Reverse	0.00	0.00, 0.00	
Parking lot-Vehicle stopped	0.00	0.00, 0.00	
Roundabout	>2,000	—	
Traffic signal-green	979	321, 2,980	
Traffic signal-red_Stop	0.00	0.00, 0.00	
**Patient**			0.5
No patient on board	—	—	
Patient on board	>2,000	—	
**II. No action required**			
**Time_of_the_day**			0.8
Day	—	—	
Night	>2,000	—	
**Status**			0.3
Available	—	—	
On scene	>2,000	—	
Refueling	0.00	0.00, 0.00	
Responding P1	108	19.7, 597	
Responding P2	>2,000	—	
Standby	>2,000	—	
Transporting_p2	663	126, 3,495	
**Type of the ambulance**			0.7
4×4	—	—	
Emergency_Rural	0.00	0.00, 0.00	
Emergency_Urban	0.00	0.00, 0.00	
Major Incident Response Truck	>2,000	—	
Non-Emergency	2.15	0.62, 7.43	
Other	0.00	0.00, 0.00	
**Location**			>0.9
Highway	—	—	
Normal road	>2,000	—	
Off-road	0.00	0.00, 0.00	
Parking lot-Driving forward	0.00	0.00, 0.00	
Parking lot-Reverse	>2,000	—	
Parking lot-Vehicle stopped	405, 409	—	
Roundabout	0.00	0.00, 0.00	
Traffic signal-green	>2,000	—	
Traffic signal-red_Stop	>2,000	—	
**Patient**			0.5
No patient on board	—	—	
Patient on board	>2,000	—	
**III. Driving refresher course for two days**			
**Time_of_the_day**			0.8
Day	—	—	
Night	>2,000	—	
**Status**			0.3
Available	—	—	
On scene	>2,000	—	
Refueling	>2,000	—	
Responding P1	>2,000	—	
Responding P2	>2,000	—	
Standby	0.01	0.01, 0.01	
Transporting_p2	>2,000	—	
**Type of the ambulance**			0.7
4×4	—	—	
Emergency_Rural	2.87	2.87, 2.87	
Emergency_Urban	>2,000	—	
Major Incident Response Truck	0.03	0.03, 0.03	
Non-Emergency	0.01	0.01, 0.01	
Other	0.01	0.01, 0.01	
**Location**			>0.9
Highway	—	—	
Normal road	>2,000	—	
Off-road	0.00	0.00, 0.00	
Parking lot-Driving forward	1.19	0.25, 5.64	
Parking lot-Reverse	0.00	0.00, 0.00	
Parking lot-Vehicle stopped	>2,000	—	
Roundabout	1.59	1.59, 1.59	
Traffic signal-green	0.00	0.00, 0.00	
Traffic signal-red_Stop	>2,000	—	
**Patient**			0.5
No patient on board	—	—	
Patient on board	0.00	0.00, 0.00	

^1^OR: Odds ratio; CI: Confidence interval. Multinomial logistic regression model accuracy: 0.41.
